# Hyaluronan Degradation by *Cemip* Regulates Host Defense against *Staphylococcus aureus* Skin Infection

**DOI:** 10.1016/j.celrep.2019.12.001

**Published:** 2020-01-07

**Authors:** Tatsuya Dokoshi, Ling-juan Zhang, Fengwu Li, Teruaki Nakatsuji, Anna Butcher, Hiroyuki Yoshida, Masayuki Shimoda, Yasunori Okada, Richard L. Gallo

**Affiliations:** 1 Department of Dermatology, University of California, San Diego, La Jolla, CA 92037, USA; 2 Biological Science Research, Kao Corporation, Odawara-shi, Kanagawa, Japan; 3 Department of Pathology, Keio University School of Medicine, Tokyo, Japan; 4 Department of Pathophysiology for Locomotive and Neoplastic Diseases, Juntendo University Graduate School of Medicine, Tokyo, Japan; 5 Lead Contact

## Abstract

*Staphylococcus aureus* is a major human bacterial pathogen responsible for deep tissue skin infections. Recent observations have suggested that rapid, localized digestion of hyaluronic acid in the extracellular matrix (ECM) of the dermis may influence bacterial invasion and tissue inflammation. In this study we find that cell migration-inducing protein (*Cemip*) is the major inducible gene responsible for hyaluronan catabolism in mice. *Cemip*^*−/−*^ mice failed to digest hyaluronan and had significantly less evidence of infection after intradermal bacterial challenge by *S. aureus.* Stabilization of large-molecular-weight hyaluronan enabled increased expression of cathelicidin antimicrobial peptide (*Camp*) that was due in part to enhanced differentiation of preadipocytes to adipocytes, as seen histologically and by increased expression of *Pref1*, *PPARg*, and *Adipoq. Cemip*^*−/−*^ mice challenged with *S. aureus* also had greater IL-6 expression and neutrophil infiltration. These observations describe a mechanism for hyaluronan in the dermal ECM to regulate tissue inflammation and host antimicrobial defense.

## In Brief

In this paper, Dokoshi et al. describe how the mammalian hyaluronidase *Cemip* is induced in the dermis during *S. aureus* infection. *Cemip* digests hyaluronan in the skin to regulate reactive adipogenesis and subsequent antimicrobial activity and skin inflammation.

## Graphical Abstract

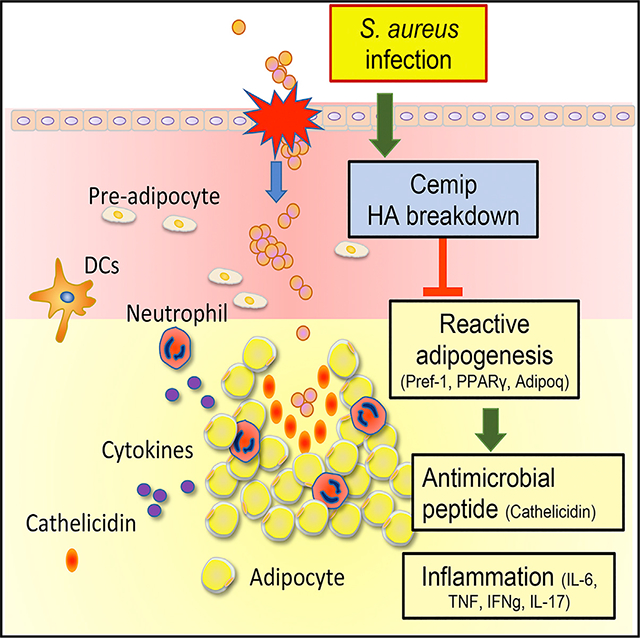

## INTRODUCTION

*Staphylococcus aureus* (*S. aureus)* and group A *Streptococcus* (GAS) are the major bacterial pathogens responsible for invasive infections of human skin. The host immune response to these pathogens remains incompletely defined. The majority of research has focused on mechanisms to limit invasion of these bacteria by the actions of resident and recruited immunocytes as well as the innate antimicrobial functions of the epidermis. However, upon disruption of the epidermal barrier, *S. aureus* or GAS encounters a very different physical environment in the dermal extracellular matrix (ECM). As a consequence, the virulence of these pathogens includes exploitation of ECM components. For example, GAS evades resident leukocyte killing by expressing long chains of hyaluronan (HA) on its surface to mimic the HA-rich ECM in the surrounding environment of the dermis (Cole et al., 2011; Wessels et al., 1991). *S. aureus* also has adapted to HA and uses its hyaluronidases to facilitate virulence (Ibberson et al., 2014, 2016). Currently, the interplay between bacterial and host HA catabolic systems has left unanswered the central question of how mammalian HA turnover during injury influences microbial resistance. In this study we sought to better understand this host response to infection.

HA is a linear polysaccharide found in the ECM of all vertebrates (Hascall et al., 2004; Toole, 1991). The functions of HA are diverse, as it is necessary for mammalian development and migration and also serves important functions in cancer and other diseases (Toole et al., 2002). Consistent with the important function of HA, the synthesis and degradation of this polysaccharide is strictly regulated and in constant dynamic equilibrium (Laurent and Fraser, 1992). A family of mammalian HA synthases and hyaluronidases are used in a cell- and tissue-specific manner to regulate tissue HA content (Erickson and Stern, 2012). Importantly, upon injury, HA is rapidly degraded, and this catabolic reaction results in important changes in the local immune response (Noble, 2002; Taylor et al., 2007a). HA fragments interact with Toll-like receptor 4 to activate cell responses during injury and have been proposed to act as a way to complement pathogen detection mechanisms (Taylor et al., 2004). Bacterial hyaluronidases such as HysA expressed by *S. aureus* degrade HA differently than the mammalian hyaluronidases and thus generate alternative products with distinct functions (Ibberson et al., 2016). However, despite the important role of HA during injury, the mechanism responsible for local regulation of HA turnover and its contribution to host defense against infection has been unknown.

Prior attempts to evaluate the function of previously defined mammalian hyaluronidases had not identified the gene responsible for mediating this critical event following infection of the skin. Cell migration-inducing protein *(Cemip),* alias HA-binding protein involved in HA depolymerization (HYBID) and KIAA1199, has been recently observed to have functional consequences in functions including deafness, bone growth, fibrosis, and tumor invasion (Shimoda et al., 2017; Tang et al., 2019; Yoshida et al., 2013; Yoshino et al., 2017, 2018). In this study we hypothesized that *Cemip* may initiate HA breakdown during deep tissue infection by *S. aureus* and could be used to address the role of HA turnover in host defense. Our observations show that *Cemip* is a critical mammalian hyaluronidase and further show how regulation of this ECM component is a key regulator of innate antimicrobial defense by the dermis.

## RESULTS

### Cemip Digests Dermal HA during *S. aureus* Skin Infection

**Figure 1.**
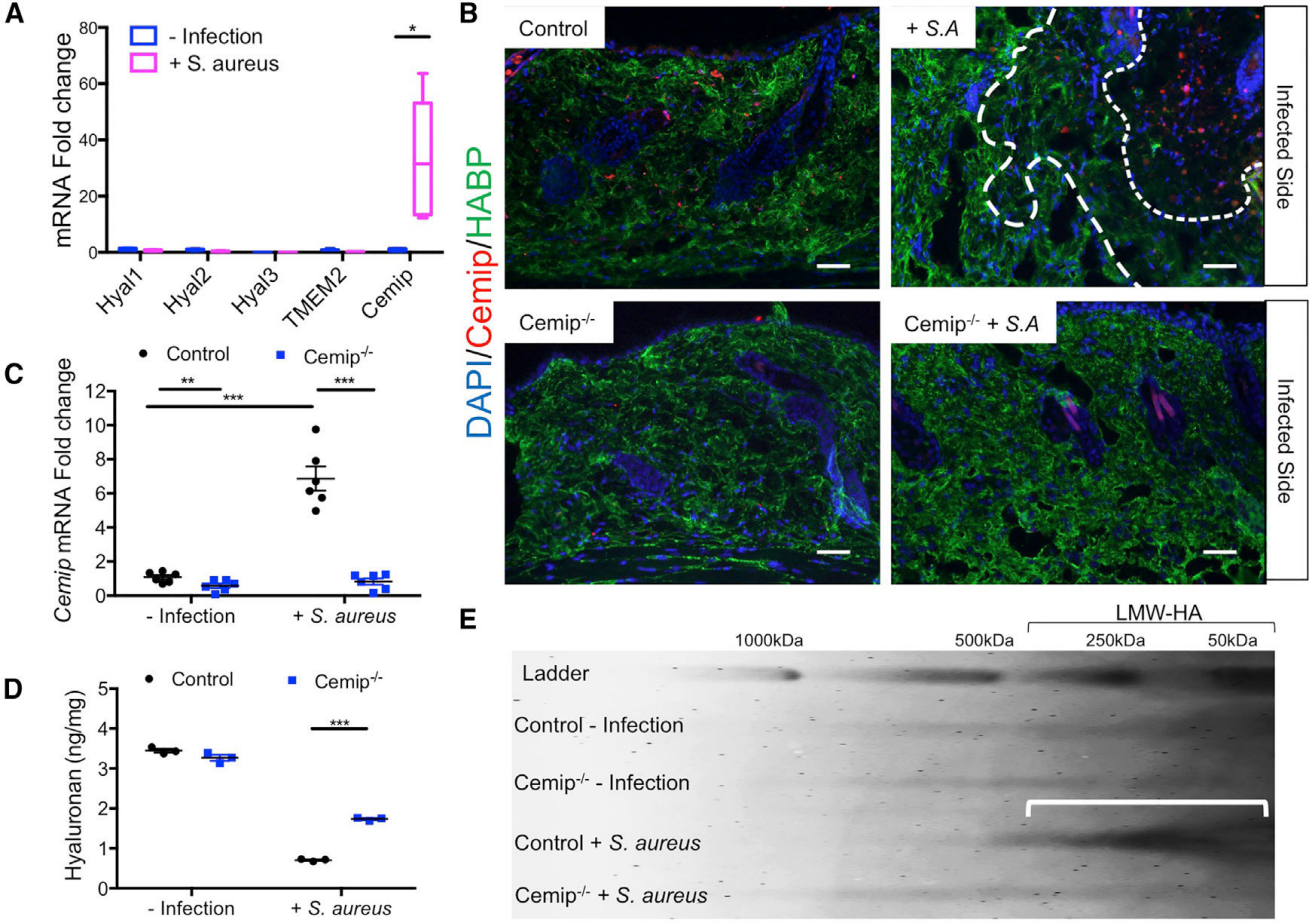
*Cemip* Is Necessary for HA Digestion after Infection (A) The expression of transcripts for five known mammalian hyaluronidases in murine skin is shown before and 3 days following infection by *S. aureus* (n = 4 control or 6 skin infection mice/group). (B) Mouse dermis stained for HA (green) or *Cemip* (red) or DAPI (blue) in representative sections of skin from control and *Cemip*^*−/−*^ mice before and 3 days after *S. aureus* infection. Dotted lines outline regions of HA loss. Infection was to the upper right in all fields shown. Scale bar, 20 μm. (C) mRNA expression from skin measured by qPCR for *Cemip* (n = 6 mice/group). (D) HA abundance measured by ELISA in skin extracts (n = 3 mice/group). (E) Gel electrophoresis and staining for HA. (a) Wild-type skin, (b) *Cemip*^*−/−*^ skin, (c) wild-type skin 3 days after *S. aureus,* and (d) *Cemip*^*−/−*^ 3 days after *S. aureus.* Arrow indicates accumulation of low-molecular weight HA as seen only in control mice after *S. aureus.* All error bars indicate mean ± SEM. *p < 0.05, **p < 0.01, and ***p < 0.001 (t test).

We examined the expression of *Cemip* in mice following inoculation of *S. aureus* into the dermis to test if this enzyme may be the hyaluronidase responsible for HA degradation during skin injury. *S. aureus* was chosen as the model skin pathogen over GAS because it does not synthesize HA itself but does produce a secreted hyaluronidase that confers virulence (Ibberson et al., 2014). *S. aureus* infection significantly increased *Cemip* mRNA in whole skin, but the expression of other murine hyaluronidases *(Hyal1, Hyal2, Hyal3,* and transmembrane protein 2 *[TMEM2])* were unchanged (Figure 1A). Immunohistochemical analysis of locally infected tissue showed that Cemip was increased in regions where HA staining was decreased (Figure 1B). *Cemip*^*−/−*^ mice failed to show an increase of *Cemip* mRNA and had a greater amount of HA in the dermis following skin infection (Figures 1B–1D). Furthermore, the decrease in the size of HA that occurs following infection was abolished in *Cemip*^*−/−*^ mice (Figure 1E, lanes c and d). Mast cell-deficient mice had less Cemip expression, a finding consistent with a role of histamine in the induction of *Cemip* (Figure S1). These observations demonstrate that *Cemip* promotes digestion of HA in the skin during infection by *S. aureus.*

### Loss of Cemip Increases Resistance against *S. aureus*

**Figure 2.**
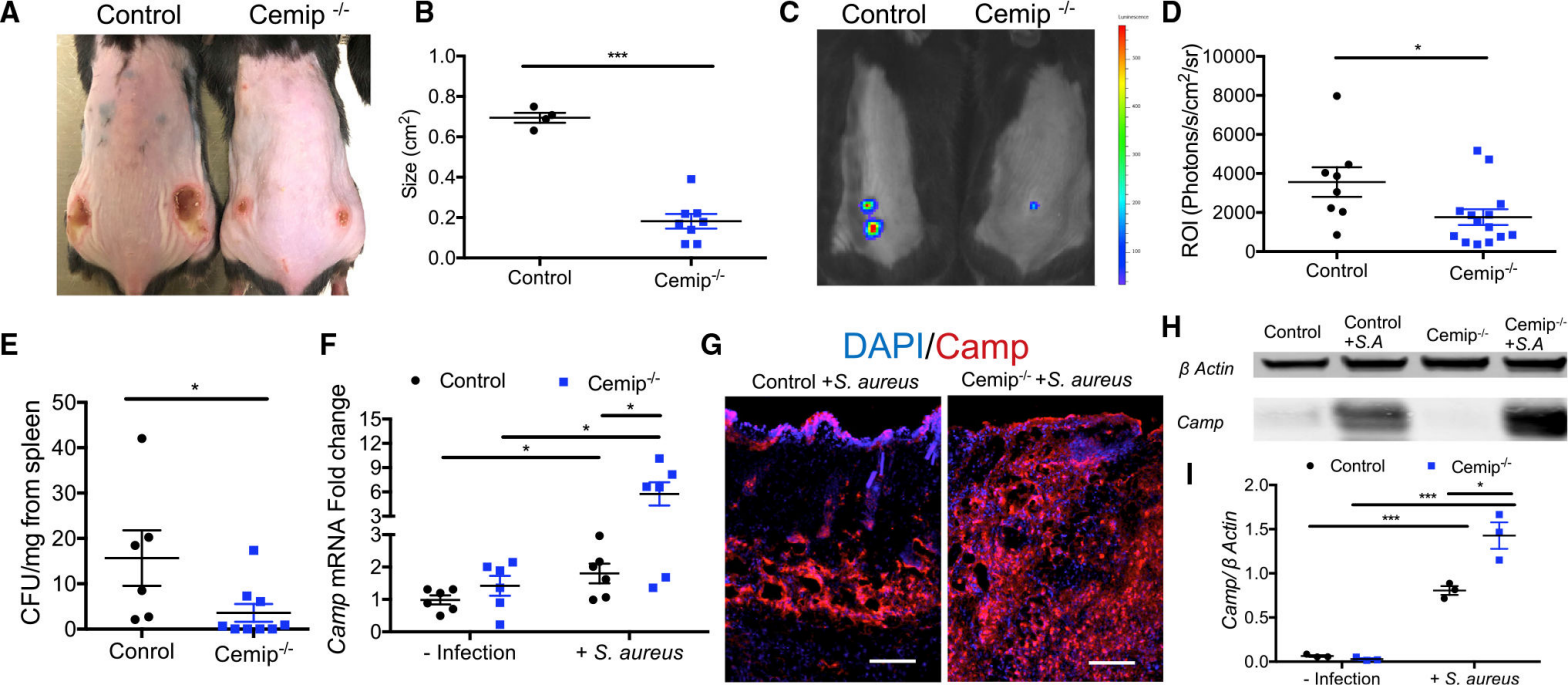
*Cemip*^*−/−*^ Mice Resist Infection by *S. aureus* (A) Skin lesions on control and *Cemip*^*−/−*^ mice 3 days after inoculation with 1 × 10^6^ colony-forming units (CFU) of *S. aureus.* (B) Measurements of lesion size on mice after inoculation with *S. aureus* as in (A). (C ) Representative images taken by IVIS. (D) Quantification of luminescence in region of interest (ROI) of skin from control and *Cemip*^*−/−*^ mice 3 days after inoculation with 1 × 10^6^ CFU of bioluminescent *S. aureus*. (E) CFU count of *S. aureus* recovered from the spleen 3 days after skin infection as in (A). (F) mRNA expression from skin measured by qPCR of *Camp* (n = 6 control and for *S. aureus*+). (G) Immunohistochemical staining for cathelicidin (red) and DAPI (blue) in representative sections of skin from control and *Cemip*^*−/−*^ mice 3 days after *S. aureus* infection. Scale bar, 50 μm. (H) Tissue extracts were subjected to immunoblotting analyses for *Camp* and beta-actin. (I) Quantification of the ratio of *Camp* to beta-actin. All error bars indicate mean ± SEM. *p < 0.05, **p < 0.01, and ***p < 0.001 (t test).

To evaluate the functional significance of HA digestion by *Cemip,* tissue injury and *S. aureus* survival were measured in the skin of *Cemip*^*−/−*^ mice. Three days after *S. aureus* injection, necrotic lesions on *Cemip*^*−/−*^ mice were significantly smaller (Figures 2A and 2B), and fewer live bacteria were evident in the skin (Figures 2C and 2D). Fewer bacteria were also detected in spleens from *Cemip*^*−/−*^ mice (Figure 2E). Because the expression of the cathelicidin antimicrobial peptide *Camp* is strongly associated with resistance to bacterial skin infections (Nizet et al., 2001), we next assessed the relative expression of *Camp* in the skin of these mice. mRNA for *Camp* was significantly increased in tissue biopsies from the infected site of *Cemip*^*−/−*^ mice (Figure 2F), and more cathelicidin protein was observed in tissue surrounding the infected area of the dermis (Figures 2G–2I). These observations suggest that loss of *Cemip* function enabled increased *Camp* expression.

### Loss of Cemip Enhances Reactive Adipogenesis

**Figure 3.**
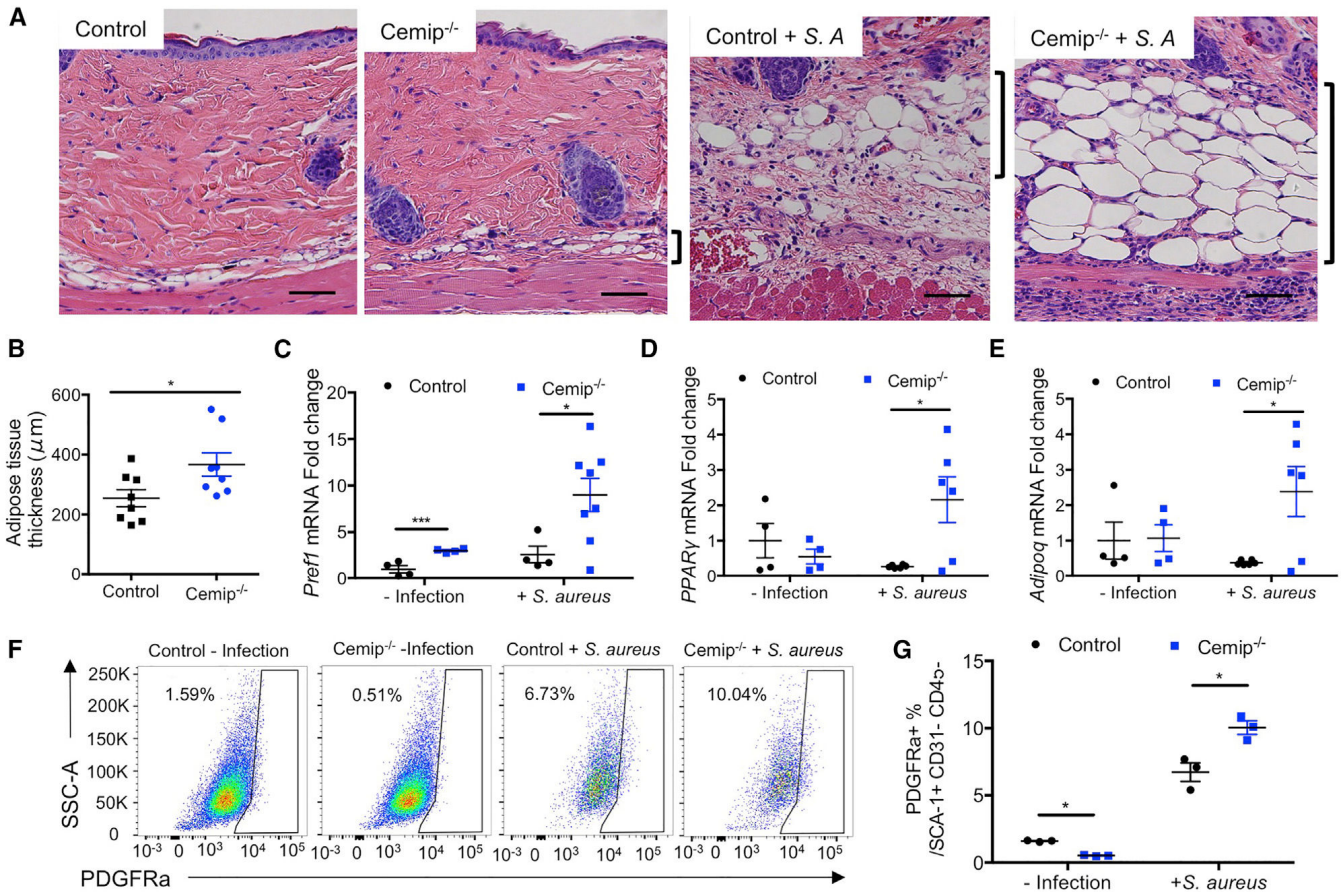
Loss of *Cemip* Enhances Reactive Adipogenesis (A) Representative histological images of skin from mice at day 0 and 3 days after infection with *S. aureus.* Tissue was stained with H&E. Brackets delineate dermal region occupied by adipocytes. Scale bar, 50 μm. (B) Quantification of the adipose tissue thickness indicated scale bar (n = 8 for control and *Cemip*^*−/−*^ infection mice/group). (C-E) qRT-PCR of the relative abundance of transcripts for (C) Pref1, (D) PPARγ, and (E) Adipoq as normalized to β-actin (n = 4 for normal condition, n = 8 for infection mice/group). (F) Flow cytometry analysis of single-cell suspensions from the skin showing expression of PEGFRα from control, *Cemip*^*−/−*^, control infection, and *Cemip*^*−/−*^ infection. Cells were gated on CD31-negative, CD45-negative, and SCA-1-positive. (G) Statistical comparison of the percentages of the cells in the indicated gates shown in (F). All error bars indicate mean ± SEM. *p < 0.05, **p < 0.01, and ***p < 0.001 (t test).

We have recently shown that a major source of cathelicidin expression in the skin comes from the local differentiation of preadipocyte fibroblasts into adipocytes, a process we refer to as reactive adipogenesis (Zhang et al., 2015). Degradation of HA inhibits the capacity of preadipocytes to differentiate into mature adipocytes (Dokoshi et al., 2018; Ji et al., 2014). Therefore, we hypothesized that the digestion of HA by *Cemip* may inhibit the local adipogenic response and thus suppress the expression of the antimicrobial peptide by these cells. Histological evaluation of the deep dermis showed a greater expansion of subcutaneous white adipose tissue (DWAT) after infection in *Cemip*^*−/−*^ mice (Figures 3A and 3B). Consistent with the observation of enhanced reactive adipogenesis by decreasing hyaluronidase activity, there was also significantly increased expression of genes associated with adipogenesis in *Cemip*^*−/−*^ mice (preadipocyte factor 1 [*Pref-1*], peroxisome proliferator-activated receptor gamma [PPARg], and adiponectin) (Figures 3C–3E). Fluorescence-activated cell sorting (FACS) analysis of skin before and after *S. aureus* infection also showed an increase in the population of preadipocytes in the dermis of *Cemip*^*−/−*^ mice as defined by CD31-negative, CD45-negative, platelet-derived growth factor receptor-α (PDGFRα)-positive, and spinocerebellar ataxia type 1 (SCA1)-positive cells (Figures 3F and 3G). Taken together, these data show that loss of Cemip results in an increase in dermal reactive adipogenesis.

### Loss of Cemip Enhances the Inflammatory Response to Infection

**Figure 4.**
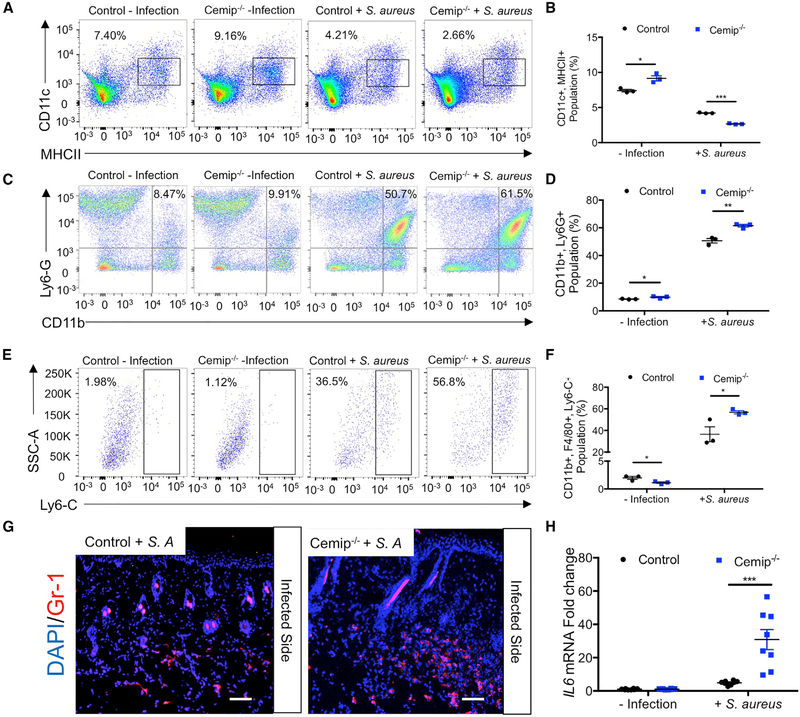
Loss of *Cemip* Enhances Local Skin Inflammation in Response to *S. aureus* (A–F) Flow cytometry analysis of single-cell suspensions from the skin showing expression of CD11c/MHCII, Ly6G/CD11b, and LyG-C cells isolated from control, *Cemip*^*−/−*^ control infection, and *Cemip*^*−/−*^ infection. Cells were gated on CD3-negative. Numbers represent the percentages of the cells in the indicated gate. (G) Representative sections of skin from control and *Cemip*^*−/−*^ mice at 3 days after *S. aureus* infection. Tissues are stained with red with Gr-1 antibody and blue with DAPI. Scale bar, 20 μm. (H) qRT-PCR of the relative abundance of transcripts for IL-6 as normalized to β-actin (n = 4 for normal condition, n = 8 for infection mice/group). All error bars indicate mean ± SEM. *p < 0.05, **p < 0.01, and ***p < 0.001 (t test).

The expression of *Camp* and other products of reactive adipogenesis can influence inflammation that may amplify the host defense against *S. aureus* (Hancock et al., 2016; Zhang et al., 2016). Therefore, we also investigated the influence of *Cemip* on resident and circulating lymphoid populations. FACS analysis of resident skin lymphoid cells revealed that *Cemip*^*−/−*^ mice had differences in the relative abundance of CD11c dendritic cells, LY6-G neutrophils, and F4/80/Ly6-C monocytes (Figures 4A–4F). Interestingly, under baseline conditions, dendritic cells and neutrophils were both slightly elevated in *Cemip*^*−/−*^ mice. Following infection, *Cemip*^*−/−*^ mice had relatively fewer dendritic cells and higher numbers of the CD11b, LY6-G, and F4/80/Ly6C positive populations. Neutrophils are a critical cell type for resistance to *S. aureus* infection, and increased numbers of LY6G-positive neutrophils were evident by immunohistochemistry (Figure 4G), and an increase in *IL-6* mRNA as measured by qPCR (Figure 4H) was detected in the skin of *S. aureus*-infected *Cemip*^*−/−*^ mice. We also evaluated systemic responses in *Cemip*^*−/−*^ mice infected by *S. aureus. Cemip*^*−/−*^ mice showed significantly lower fractions of T-box transcription factor (Tbet)+, retinoid-related orphan receptor gamma T (RORgt)+, interferon gamma (IFNg)+, and IL-17+ T cells in the spleen (Figures S2A–S2F). Overall, the loss of *Cemip* expression resulted in an enhanced local inflammatory response and decreased systemic inflammatory response after *S. aureus* infection.

## DISCUSSION

The results of this study highlight the recently appreciated role of the ECM and resident, non-lymphoid cells in the dermis to play an important role in host defense against bacterial infection. Large-molecular weight HA is highly abundant and is the major component of the ECM (Tammi et al., 1994). HA digestion into small fragments after injury has been shown to have important implications for inflammatory responses *in vivo* (Jiang et al., 2005; Muto et al., 2014; Noble et al., 1996; Taylor et al., 2004) and can modify infection by GAS through digestion of the HA-rich bacterial capsule of this organism (Schommer et al., 2014). However, a clear understanding of the mechanism responsible for HA catabolism or its connection to host antimicrobial defense has not been previously defined. In this study, we show that *Cemip* is responsible for the increase in endogenous hyaluronidase activity seen during deep tissue infection. These observations provide important new insight into the mechanisms that function in the dermis to resist invasive *S. aureus* infection.

Several complementary experimental observations made here support the conclusion that *Cemip* digests HA during skin infection. These include observations of increased transcript abundance, increased protein abundance, and decreased large–molecular weight HA corresponding to the timing, localization, and hyaluronidase activity of *Cemip.* There was no evidence of an increase in the expression of other hyaluronidases such as *Hyal1, Hyal2, Hyal3,* and *TMEM2.* HYAL4 has chondroitinase, not hyaluronidase activity (Kaneiwa et al., 2012), and expression of PH-20 is restricted to testes (Cherr et al., 1996), and these were therefore not examined. A loss of hyaluronidase activity was apparent in *Cemip*^*−/−*^ mice, as they did not show the decrease in the size of HA after infection that was observed in controls, and they had less loss of total large–molecular weight HA as measured by staining with HA-binding protein or ELISA. It has also been suggested that reactive oxygen species are also involved in HA degradation after tissue injury (Bates et al., 1984). Our observations do not exclude this as an additional mechanism, or that other enzymes contributed by the host or the bacteria themselves could be also contributing to the turnover in HA. On the contrary, as it has been estimated that an adult human contains 15 g of HA and that about one-third turns over daily (Tammi et al., 1991), it is very likely that other hyaluronidases are participating in the steady-state turnover of HA. Furthermore, *S. aureus* itself can contribute hyaluronidase activity to the site of infection through expression of *HysA,* an enzyme secreted by the pathogen and associated with virulence (Ibberson et al., 2014). The findings of this report to not exclude contribution of other enzymes to balance of HA but do clearly show that *Cemip* is responsible for a major fraction of the local increase in HA breakdown that occurs during *S. aureus* infection.

Further studies are required to better define the cell of origin of *Cemip,* which originally was discovered in dermal fibroblasts. In preliminary experiments, we have performed single-cell RNA sequencing (RNA-seq) of whole tissue and *in vitro* analysis of potential candidate cell types that may express *Cemip*. These studies have not yet convincingly defined a primary cell type responsible for its synthesis but suggest that a fibroblast cell type may be the origin. *Cemip* has also been shown to be induced by histamine (Yoshino et al., 2018), and as histamine in skin is primarily released by mast cells, we examined if these cells could contribute to the response we observed. Mast cell-deficient mice had some-what less *Cemip* but were still able to show increased expression after infection (Figure S2).

The most direct explanation for increased resistance to *S. aureus* in *Cemip*^*−/−*^ mice is the increase in *Camp* produced by the rapid, local differentiation of preadipocyte fibroblasts to mature fat (Zhang et al., 2015). A persistence of high–molecular weight HA enables this adipogenic response and results in much greater expression of *Camp* in the dermis at the site of infection. We have previously shown that increased hyaluronidase-1 activity (in contrast with the loss of hyaluronidase activity seen here in *Cemip*^*−/−*^ mice) will inhibit reactive adipogenesis and *Camp* expression (Dokoshi et al., 2018). Our findings described in Figures 2 and 3 show that loss of *Cemip* enables the skin to respond to *S. aureus* infection by increasing expression of *Camp*, expanding DWAT and enhancing gene expression associated with adipogenesis.

The present observations suggest that local induction of hyaluronidase activity has a negative consequence to the host, as it enables greater bacterial proliferation and infection than when *Cemip* is deleted. Why then has this activity been maintained? We speculate that the presence of inducible hyaluronidase activity serves two complementary purposes. First, prior work has shown that low–molecular weight fragments of HA generated by hyaluronidases are potent danger-associated molecular patterns (DAMPs) and serve to alert the host of injury even under aseptic conditions (Mummert, 2005; Taylor et al., 2007b; Yamasaki et al., 2009). Consistent with the systemic alarmin function of HA fragments as a DAMP, we observed a greatly decreased induction of CD4+ cells expressing Tbet, GATA3, RORγt, INFγ, and1 IL-17 in *Cemip*^*−/−*^ mice following infection. Thus, *Cemip* may provide one of many systemic signals of injury. A second beneficial consequence of transient, inducible hyaluronidase activity is that the digestion of HA inhibits excess local inflammatory responses. Hyaluronidase expression can inhibit antigen presentation (Muto et al., 2014), thus potentially preventing unwanted allergic sensitization to common antigens that become accessible during injury. *Cemip*^*−/−*^ mice studied here had greater IL-6 and neutrophil infiltration, a reaction that may have helped fight infection. However, the immunological consequences to infection in *Cemip*^*−/−*^ mice are complicated, as a lower bacterial burden and higher *Camp* expression can indirectly alter local innate and adaptive immune responses. Furthermore, HA itself influences many different aspects of cell differentiation and migration (West et al., 1985). Major clinical developmental phenotypes result from mutations in hyaluronidases such as mucopolysaccharidosis type IX from *Hyal1* (Natowicz et al., 1996), bone defects, and cardiopulmonary dysfunction from loss of *Hyal2* (Jadin et al., 2008; Chowdhury et al., 2013). Deafness and other abnormalities are associated with defects in *HYBID* (Shimoda et al., 2017; Tang et al., 2019; Yoshida et al., 2013; Yoshino et al., 2017, 2018). Thus, as is frequently the case, this gene has several essential functions that extend beyond the immune defense role we have defined here. Past and current findings suggest that the influence of HA turnover on host defense functions is complex and likely acts in multiple ways beyond direct antimicrobial activity.

In summary, this work has solved the long unanswered question of what induces digestion of HA in the dermis after infection. This further clarifies the molecular steps necessary for the dermis to resist deep tissue infection by *S. aureus.* HA is an essential component of ECM of many organs and often exploited by microbes through molecular mimicry. The fundamental roles of elements of the ECM in immune defense are specific to context and may vary by organ and microorganism. Understanding how the skin initiates the digestion of HA can have important diagnostic and therapeutic implications for many infectious and inflammatory diseases.

## STAR★METHODS

### LEAD CONTACT AND MATERIALS AVAILABILITY

This study did not generate new unique reagents. Further information and requests for plasmids, resources, and reagents should be directed to and will be fulfilled by the Lead Contact, Richard L Gallo (rgallo@ucsd.edu).

### EXPERIMENTAL MODEL AND SUBJECT DETAILS

#### Animals and animal care

Cemip (KIAA1199) KO mice were generated using a gene targeting Cre-loxP system as described in the other report (Shimoda et al., 2017; Yoshino et al., 2017). Wild-type mice (C57BL/6 mice) and K14-cre transgenic mice were obtained from The Jackson Laboratory. *Scf*.^fl/fl^ mice (a gift from Dr. Dinald at University of California San Diego). K14-cre transgenic mice were bred with *Scf*.^fl/fl^ mice for the generation of K14-cre *Scf*.^fl/fl^ mice. K14-cre littermate controls were used in all experiments. All animal experiments were approved by the University of California, San Diego, Institutional Animal Care and Use committee. For all animal studies, animals were randomly selected without formal pre-randomization and quantitative measurements were done without the opportunity for bias.

#### Bacterial strains

*S. aureus* strain USA300 is a predominant community-associated Methicillin-resistant *S. aureus* (MRSA) strain and AH4807, a USA300 MRSA strain was tested in a manner that was similar to previously described (Muhs et al., 2017; Paharik et al., 2017), was kindly provided by Alexander Horswill (Deprtment of Immunology & Microbiology at the University of Colorado).

#### Mouse model of S. aureus skin infection

Skin infection experiments were done as described before (Nizet et al., 2001). *S. aureus* strain USA300 was used for infection. In brief, the backs of sex-matched and age-matched (8 week to 12 week) adult wild-type or Ella/Hyal1 mice were shaved and hair removed by chemical depilation (Nair) then injected subcutaneously with 100 μL of a mid-logarithmic growth phase of S. aureus (2× 10^6^ CFU of bacteria) in PBS. Mice were sacrificed after day 3 and 8 mm skin punch biopsy comprising the center of the injection site was harvested. Infected skin surrounding the infection center (6–8 mm) void of center abscess was carefully dissected out for RNA extraction or CFU determination. Skin biopsies were homogenized in 1 mL Trizol (for RNA) or PBS (for CFU counting) with 2 mm zirconia beads in a mini-bead beater 16 (Biospect, Bartlesville, OK). To count CFU, homogenized skin samples were serially diluted, plated onto Tryptic Soy Agar, and enumerated after 18 hours to quantify the CFU per gram of tissue. For *in vivo* live bacterial imaging, mice were imaged under isoflurane inhalation anesthesia (2%). Photons emitted from luminescent bacteria were collected during a 1 min exposure using the Xenogen IVIS Imaging System and living image software (Xenogen, Alameda, CA). Bioluminescent image data are presented on a pseudocolor scale (blue representing least intense and red representing the most intense signal) overlaid onto a gray-scale photographic image. Using the image analysis tools in living image software, circular analysis windows (of uniform area) were overlaid onto regions of interest and the corresponding bioluminescence values (total flux) were measured.

#### Study approval

All animal experiments were approved by the University of California, San Diego, Institutional Animal Care and Use committee. For all animal studies, animals were randomly selected without formal pre-randomization and quantitative measurements were done without the opportunity for bias.

### METHOD DETAILS

#### Chemicals and reagents

##### Rat anti-Cemip antibodies were provided by KAO company

Rabbit anti-CAMP antibodies were made from our lab as described previously (Dorschner et al., 2001); rabbit anti-PREF1/DLK antibodies are from Abcam (Cambridge, MA); BODIPY® FL dye was purchased from Thermo Fisher (Houston, TX). HA binding protein was purchased from Millipore., mouse-Hyal1, Hyal2, KIAA1199, TMEM2, HAS1, HAS2, HAS3, ZFP423, Pref1, PPARg, Adipoq, CEBPA, CAMP, IL6, TNF Taqman gene expression assay were purchased from Life Technologies Corporation (Grand Island, NY).

#### Reverse transcription-quantitative PCR (RTqPCR) analyses

RTqPCR was used to determine the mRNA abundance as described previously (Morioka et al., 2008). Total cellular RNA was extracted using the PureLink RNA Mini Kit (Life Technologies Corporation, Grand Island, NY) and mRNA were purified by using Dynabeads mRNA Purification Kit(Life technologies). 100 ng of mRNA was reverse transcribed to cDNA using iScript cDNA synthesis kit (Bio-Rad Laboratiries, Inc. Hercules, CA). Quantitative, real-time PCR was performed on the CFX96 real time system (Biorad) using predeveloped Taqman gene expression assay (Applied Biosystems). The expression of β-Actin gene was used as a house keeping gene to normalize data.

#### Histology and immunohistochemistry (IHC)

Tissue biopsies were directly embedded in OCT compound or paraffin. Paraffin embedded tissues are used for Hematoxylin/Eosin (H&E) staining, and frozen sections were fixed in 4% PFA for 20 mins or 100% acetone prior to immunofluorescence staining. For IHC, fixed and permeabilized frozen tissue sections were blocked with Image-iT FX reagent (Invitrogen) before incubating with primary antibodies followed by appropriate 488- or 568-coupled secondary antibodies. Nuclei were counterstained with DAPI. All images were taken with an Olympus BX41 microscope (widefield) or Zeiss LSM510 confocal microscope as indicated.

#### Flow cytometry analyses

Colon collected from control or DSS-treated mice was cut into small pieces then digested with 2.5 mg/mL Collagenase D and 30 ng/mL DNase1 for 2 hours at 37°C then filtered through a 30 μm filter to generate single cell suspension for FACS analyses. Cells were then stained with zombie violet viability dye (BioLegend, 423114), blocked with anti-mouse CD16/32 (eBioscience, 14016185), followed by staining with antibody cocktails for preadipocytes or immune cells. The antibody cocktail for preadipocytes includes AF488-SMA (eBioscience, 53976082), PECy7-CD45 (BioLegend, 147704), PerCy5.5-CD31 (BioLegend, 102522), PE-Thy1 (BioLegend, 105308), APC-PDGFRa (eBioscience, 17140181), BV605-SCA1 (BioLegend, 108133) and AF700-CD24 (BioLegend, 108136). The antibody cocktail for immune cells includes PECy7-CD11b (BioLegend, 101216), FITC-Ly6G (eBioscience, 11593182), PE-F4/80 (eBioscience,12480182), APC-CD11C (BioLegend, 117310), AF700-MHCII (eBioscience, 56532182), APC-Cy7-CD3 (BioLegend, 100222), Tbet (Fisher Scientific, 562467), GATA3(BioLegend, 653807), RORgt (eBioscience, 12–6981-80), IFN-gamma (BioLegend, 505809), IL-17 (BioLegend, 506929), Foxp3 (eBioscience, 48–5773-80) and Fixable Viability Dye eFluor 506 (eBioscience, 65–0866-14)FACS analyses for surface expression of preadipocyte or immune cell markers were performed by the BD FACSCanto RUO machine and analyzed by FlowJo V10 software. Dead cells stained positive with zombie violet dye were excluded from the analyses.

#### Hyaluronan (HA) analysis

Glycosaminoglycan (GAGs), including HA were extracted from murine skin as previously described (Muto et al., 2014).Samples were homogenized and treated overnight with protease (0.16 mg/ml; Sigma-Aldrich) to degrade protein, followed by purification by anion exchange chromatography using DEAE Sephacel (Amersham Biosciences). Columns were washed with a low-salt buffer (0.15 M NaCl in 20 mM sodium acetate; pH 6.0) and eluted with 1 M NaCl. Glycans were desalted by PD10 (GE Healthcare). HA concentrations were measured ELISA Duo Set (R&D Systems). The size distribution of HA was analyzed by agarose gel electrophoresis (Lee and Cowman, 1994). The HA sample was mixed with TAE buffer containing 2 M sucrose and electrophoresed at 2 V/cm for 10 hours at room temperature. The gel was stained overnight under light-protective cover at room temperature in a solution containing 0.005% Stains-All in 50% ethanol, and destained in water. Hyalose ladders (Hyalose) were used for standards.

### QUANTIFICATION AND STATISTICAL ANALYSIS

Experiments were repeated at least three times with similar results. Statistical significance was determined using Student’s unpaired two-tailed t test, or one-way ANOVA multiple comparison test as indicated in the legend (*p < 0.05, **p < 0.01, ***p < 0.001).

### DATA AND CODE AVAILABILITY

The published article includes all datasets generated or analyzed during this study.

## Supplementary Material

1

2

## Figures and Tables

**KEY RESOURCES TABLE:** 

REAGENT or RESOURCE	SOURCE	IDENTIFIER
Antibodies		
Anti-CAMP	(Dorschner et al., 2001)	N/A
Anti-PREF1/DLK	Abcam	Cat# ab21682
BODIPY®	Thermo Fisher	Cat# A-577
Anti-HA binding protein	Millipore	Cat# 385910
Anti-Cemip	(Shimoda et al., 2017)	N/A
zombie violet viability dye	BioLegend	Cat# 423114
Anti-mouse CD16/32	eBioscience	Cat# 14016185
Anti-mouse SMA	eBioscience	Cat# 53976082
Anti-mouse CD45	BioLegend	Cat# 147704
Anti-mouse CD31	BioLegend	Cat# 102522
Anti-mouse Thy1	BioLegend	Cat# 105308
Anti-mouse PDGFRa	eBioscience	Cat# 17140181
Anti-mouse SCA1	BioLegend	Cat# 108133
Anti-mouse CD24	BioLegend	Cat# 108136
Anti-mouse CD11b	BioLegend	Cat# 101216
Anti-mouse Ly6G	eBioscience	Cat# 11593182
Anti-mouse F4/80	eBioscience	Cat# 12480182
Anti-mouse CD11C	BioLegend	Cat# 117310
Anti-mouse MHCII	eBioscience	Cat# 56532182
Anti-mouse CD3	BioLegend	Cat# 100222
Anti-mouse Tbet	Fisher Scientific	Cat# 562467
Anti-mouse GATA3	BioLegend	Cat# 653807
Anti-mouse RORgt	eBioscience	Cat# 12–6981-80
Anti-mouse IFN-gamma	BioLegend	Cat# 505809
Anti-mouse IL-17	BioLegend	Cat# 506929
Anti-mouse Foxp3	eBioscience	Cat# 48–5773-80
Bacterial and Virus Strains		
S. *aureus* strain USA300	(Muhs et al., 2017)	N/A
Chemicals, Peptides, and Recombinant Proteins		
protease	Sigma-Aldrich	Cat# P1236
DEAE Sephacel	Amersham Biosciences	Cat# 17070901
Prepacked Disposable PD-10 Columns	GE Healthcare	Cat# 17085101
Hyalose ladders HiLadder	Hyalose	Cat# HYA-HILAD-20
Critical Commercial Assays		
DuoSet ELISA Ancillary Reagent Kit	R&D Systems	Cat# DY007
iScript cDNA synthesis kit	Bio-Rad	Cat# 1708890
PureLink RNA Mini Kit	Life Technologies	Cat# 12183025
Experimental Models: Organisms/Strains		
Mouse:C57BL/6J	Jackson Laboratory	Stock No: 000664
Mouse:Cemip (KIAA1199) KO	KAO (Shimoda et al., 2017)	N/A
Mouse:S*cf.*^fl/fl^	Di Nardo Lab (Wang et al., 2017)	N/A
Mouse:B6N.Cg-Tg(KRT14-cre)1Amc/J	Jackson Laboratory	Stock No: 018964
Oligonucleotides		
Actb	ThermoFisher	Mm02619580_g1
Hyal1	ThermoFisher	Mm00476206_m1
Hyal2	ThermoFisher	Mm01230688_g1
Hyal3	ThermoFisher	Mm00662097_m1
KIAA1199	ThermoFisher	Mm00472921_m1
TMEM2	ThermoFisher	Mm00459599_m1
HAS1	ThermoFisher	Mm03048195_m1
HAS2	ThermoFisher	Mm00515089_m1
HAS3	ThermoFisher	Mm00515092_m1
ZFP423	ThermoFisher	Mm00677660_m1
Pref1	ThermoFisher	Mm00494477_m1
PPARg	ThermoFisher	Mm00440940_m1
Adipoq	ThermoFisher	Mm00456425_m1
CEBPA	ThermoFisher	Mm00514283_s1
CAMP	ThermoFisher	Mm00438285_m1
IL6	ThermoFisher	Mm00446190_m1
TNF	ThermoFisher	Mm00443258_m1
Software and Algorithms		
FlowJo	FlowJo	https://www.flowjo.com/
IVIS living image software	Xenogen	https://www.perkinelmer.com/
Prism	GraphPad Software	https://www.graphpad.com/


## Highlights

- *Cemip* is induced following skin infection and digests hyaluronan
- Hyaluronan is required for optimal reactive adipogenesis and antimicrobial activity
- Digestion of hyaluronan by *Cemip* inhibits reactive adipogenesis
- *Cemip* loss increases inflammation and antimicrobial activity following skin infection
