# Supplementary material for: Hyaluronan Degradation by Cemip Regulates Host Defense against Staphylococcus aureus Skin Infection
Source: Cell Rep. Author manuscript; Available in PMC 2020 Feb 19. (PMC7029423; doi:10.1016/j.celrep.2019.12.001)
Supplement: 1 [file NIHMS1548566-supplement-1.pdf]

Cell Reports, Volume *30*

Supplemental Information

Hyaluronan Degradation by *Cemip* Regulates Host Defense against *Staphylococcus aureus* Skin Infection

Tatsuya Dokoshi, Ling-juan Zhang, Fengwu Li, Teruaki Nakatsuji, Anna Butcher, Hiroyuki Yoshida, Masayuki Shimoda, Yasunori Okada, and Richard L. Gallo

Supplemental Figures

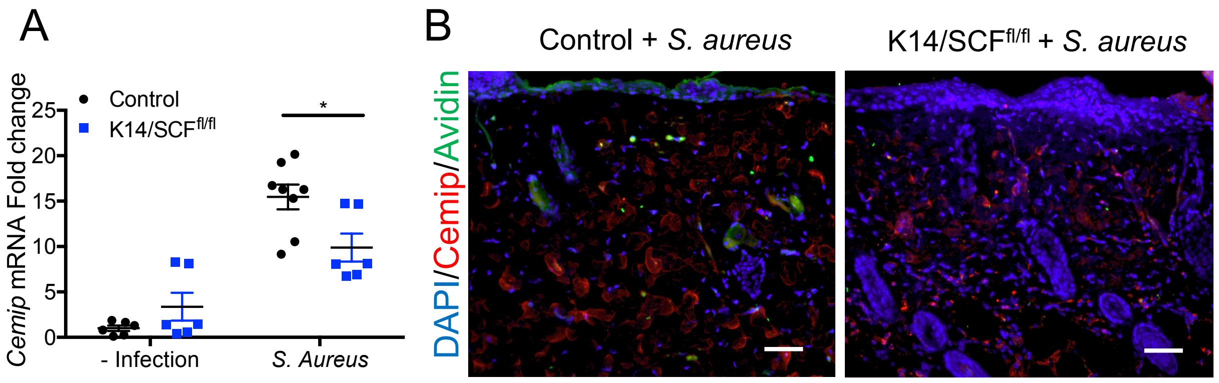

Supplemental Figure 1. *Cemip* expression in mast cell deficient mice. Related to figure 1.

(**A**) mRNA expression from skin measured by qPCR of *Cemip* (n=6 mice/group). (**B**) Mouse dermis stained for Avidin (green) or *Cemip* (red) or DAPI (blue) in representative sections of skin from control and K14/SCF$^{fl/fl}$ mice 3 days after *S. aureus* infection. Dotted lines outline regions of HA loss. Scale Bar = 20 Microns. All error bars indicate mean ± SEM; * P<0.05, ** P < 0.01, *** P<0.001 (t test).

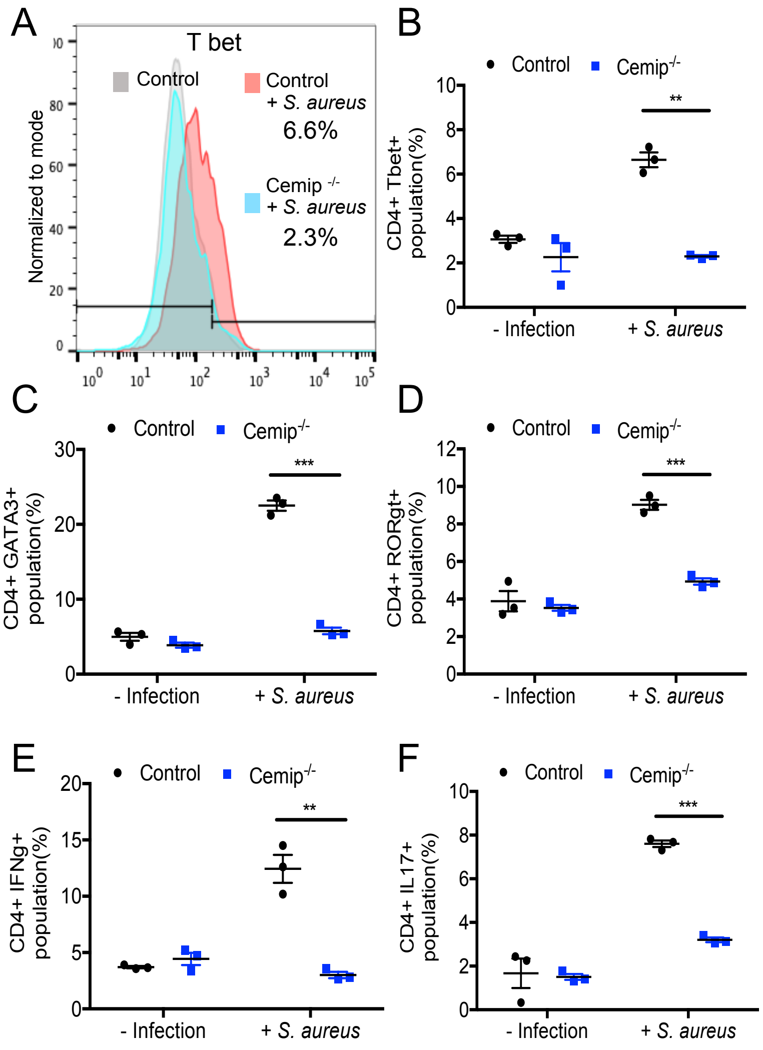

Supplemental Figure 2. Loss of *Cemip* decreases the systemic response to *S. aureus.* Related to Figure 4.

(A to F) Flow cytometry analysis of single cell suspensions from the spleen showing expression of Tbet, GATA3, RORγt, INFγ and1 IL-17 from control, *Cemip*$^{-/-}$, Control with *S. aureus* infection and *Cemip*$^{-/-}$ *with S. aureus* infection. Cells were gated on CD4 positive. Numbers represent the percentage of the cells in the indicated gate. (n=3) All error bars indicate mean ± SEM; * P<0.05, ** P < 0.01, *** P<0.001 (t test).
